# Randomized Pharmacokinetic Study Comparing Subcutaneous and Intravenous Palonosetron in Cancer Patients Treated with Platinum Based Chemotherapy

**DOI:** 10.1371/journal.pone.0089747

**Published:** 2014-02-27

**Authors:** Belen Sadaba, Anabel del Barrio, Miguel Angel Campanero, Jose Ramon Azanza, Almudena Gomez-Guiu, Jose Maria Lopez-Picazo, Salvador Martin Algarra, Francisco Guillén Grimá, Maria Blanco Prieto, Jose Luis Perez-Gracia, Alfonso Gurpide

**Affiliations:** 1 Clinical Pharmacology Department, School of Pharmacy, Clinica Universidad Navarra, University of Navarra, Pamplona, Spain; 2 Oncology Department, School of Pharmacy, Clinica Universidad Navarra, University of Navarra, Pamplona, Spain; 3 Department of Preventive Medicine, School of Pharmacy, Clinica Universidad Navarra, University of Navarra, Pamplona, Spain; 4 Department of Pharmacy and Pharmaceutical Technology, School of Pharmacy, Clinica Universidad Navarra, University of Navarra, Pamplona, Spain; Yale University, United States of America

## Abstract

**Background:**

Palonosetron is a potent second generation 5- hydroxytryptamine-3 selective antagonist which can be administered by either intravenous (IV) or oral routes, but subcutaneous (SC) administration of palonosetron has never been studied, even though it could have useful clinical applications. In this study, we evaluate the bioavailability of SC palonosetron.

**Patients and Methods:**

Patients treated with platinum-based chemotherapy were randomized to receive SC or IV palonosetron, followed by the alternative route in a crossover manner, during the first two cycles of chemotherapy. Blood samples were collected at baseline and 10, 15, 30, 45, 60, 90 minutes and 2, 3, 4, 6, 8, 12 and 24 h after palonosetron administration. Urine was collected during 12 hours following palonosetron. We compared pharmacokinetic parameters including AUC_0–24h_, t_1/2_, and C_max_ observed with each route of administration by analysis of variance (ANOVA).

**Results:**

From October 2009 to July 2010, 25 evaluable patients were included. AUC0–24h for IV and SC palonosetron were respectively 14.1 and 12.7 ng × h/ml (p = 0.160). Bioavalability of SC palonosetron was 118% (95% IC: 69–168). C_max_ was lower with SC than with IV route and was reached 15 minutes following SC administration.

**Conclusions:**

Palonosetron bioavailability was similar when administered by either SC or IV route. This new route of administration might be specially useful for outpatient management of emesis and for administration of oral chemotherapy.

***Trial Registration*:**

ClinicalTrials.gov NCT01046240

## Introduction

Emesis remains one of the most relevant side effects of chemotherapy. It induces a decrease in health-related quality of life and it is often underestimated by physicians [1], [2]. 5-hydroxytryptamine-3 (5-HT3) inhibitors are universally recommended as part of standard anti-emetic premedication for moderate and highly emetogenic chemotherapy agents [3], [4]. Palonosetron (Aloxi; Italfarmaco Laboratories,) is a potent and highly selective 5-HT3 inhibitor with a prolonged half-life (40 hours), which has up to 30 times higher affinity for the receptor than first-generation 5-HT3 antagonists. In addition, it has weak antagonistic action against other 5-HT receptors [5]. The efficacy of palonosetron in the prevention of nausea and vomiting has been shown in several phase III studies [6]–[8].

Palonosetron, as the other 5-HT3 antagonists, can be administered by oral or intravenous (IV) route. However, these routes are inadequate for patients managed in the outpatient setting that cannot tolerate oral medication, due to vomiting or other reasons. Subcutaneous (SC) administration of palonosetron could be an attractive option for these patients and for those that receive oral chemotherapy and do not require an intravenous access. Theoretical advantages of SC route over IV delivery include its simpler administration, as well as its decreased complications and costs. In a previous study, we compared the administration of SC and IV granisetron and we found that both administration routes have similar bioavailability [9]. The objective of this study was to compare the bioavailability of SC and IV palonosetron, in order to establish the validity of SC administration for cancer patients. We performed a pharmacokinetic evaluation of SC and IV palonosetron, using a randomized crossover design. We hypothesized that bioavailability of SC palonosetron would not be inferior to that achieved by IV delivery.

## Patients and Methods

Eligible patients had to be candidates to receive platinum-based chemotherapy. Additional inclusion criteria were: adequate bone marrow, renal and hepatic function, respectively defined by: absolute neutrophil count ≥1500/mm^3^ and platelets ≥100000/mm^3^; creatinine<1.5 mg/dl; and bilirubin, AST and ALT≤2 times x upper limit of normality. Patients must had ECOG performance status ≤2. Patients were not eligible in case of pregnancy or relevant concomitant diseases.

Chemotherapy was the same in both cycles for each patient. Patients were randomized to receive SC or IV palonosetron 250 µg during the first cycle and to crossover to the alternative route during the second one. For IV treatment, 250 µg of palonosetron were injected over 30 seconds. For SC treatment 250 µg of palonosetron were administered subcutaneously in the abdomen. Patients received 20 mg of intravenous dexamethasone and further anti-emetic treatment if necessary, although no additional doses of palonosetron were administered, to avoid pharmacokinetic interference. The protocol for this trial and supporting CONSORT checklist are available as supporting information; see Checklist S1 and Protocol S1.

The main endpoint was bioavailability (F). Even though the study was not designed to test clinical efficacy, patients evaluated their emetic symptoms by completing a diary. Toxicity was assessed using Common Toxicity Criteria for adverse events (CTCAE) version 3.0. (http://ctep.cancer.gov/protocoldevelopment/electronic_applications/docs/ctcaev3.pdf).

All patients signed written informed consent before treatment. The protocol was approved by the Clinical Research Ethics Committee of Navarra and by the Spanish Agency for Medicines and Healthcare Products. The trial was registered in ClinicalTrials.gov (NCT01046240, URL: http://clinicaltrials.gov/ct2/show/NCT01046240?term=palonosetron+sadaba&rank=1).

### Pharmacokinetic study

Blood samples (5 ml) were obtained at baseline (pre-dose), 10, 15, 30, 45, 60 minutes and 1.5, 2, 3, 4, 6, 8, 12 and 24 hours following administration of palonosetron. Blood was drawn in heparin tubes, centrifuged (4°C, 3500 r.p.m., 10 minutes) and frozen at −20°C until analysis. Urine was collected for 12 hours after treatment. Palonosetron levels were determined by a validated high performance liquid chromatography with mass/mass detection after liquid/liquid extraction of acidified plasma samples. The quantitation limit was 0.1 ng/ml. Calibration curves were prepared at a concentration range of 0. 1–100 ng/ml. Plasma concentrations were analyzed by a laboratory certified in Good Laboratory Practices.

Pharmacokinetic parameters were calculated by noncompartimental methods. All calculations were carried out using WinNonlin Professional Version 5.3 (Scientific Consulting, Inc., Mountain View, USA). AUC_0–12h_ and AUC_0–24h_ were calculated by the trapezoidal rule. Maximum concentration (C_max_) and time to maximum concentration (t_max_) were obtained from experimental data. Half-life (t_1/2_) and terminal phase rate constant (k_e_) were determined by unweighted non-linear regression analysis of the terminal slope of the log-plasma concentration-time curve.

### Statistical analysis

Twenty-five patients were required to have a power of 0.80 in order to conclude equivalence at the significance level 0.05 in total bioavailability of SC administration in relation to IV administration. We compared pharmacokinetic parameters by analysis of variance (ANOVA) including the factors sequence, period, formulation and study participant to the log-transformed parameters log(AUC) and log(C_max_). We estimated the relative bioavailability and the 90% confidence intervals (CIs) by the residual variance of the ANOVA [10]. Other pharmacokinetic parameters were analyzed by paired Student's t test or Wilcoxon test. Statistical analysis was performed using SPSS 15.0 and WinNonlin Pro 5.3. The emetic symptoms were compared by McNemar's test. The 95% Cis for proportions were calculated using Epiinfo 6.11.

## Results

From October 2009 to July 2010, 25 evaluable patients were included. Four additional patients were not evaluable because of anaphylactic shock during administration of paclitaxel (1), volunteer decision to leave the study (1), death due to disease progression (1) and chemotherapy related neutropenia (1). Patient characteristics are described in table 1. Gender distribution was 18 male (72%) and 7 female (28%). Mean age was 58 years (SD = 12.4) and mean body mass index 27.2 kg/m^2^ (SD = 4.7).

**Table 1:** Patient characteristics.

	N	%	Mean	Range
**Patients**	25	-	-	-
**Age (years)**	-	-	58	31-74
**Sex**				
** Male**	18	72		
** Female**	7	28	-	-
**Weight (kg)**	-	-	77	50.8-121
**Height (cm)**	-	-	167.6	153-182
**Body mass index (kg/m^2^)**	-	-	27.2	19.2-38.1
**ECOG**				
** 0**	10	40	-	-
** 1**	12	48	-	-
** 2**	3	12	-	-
**Tumours**				
** NSCLC stage IV**	12	48	-	-
** SCLC**	2	8	-	-
** Bladder cancer**	5	20	-	-
** Pelvis kidney cancer**	1	4	-	-
** Tongue cancer**	1	4	-	-
** Nasopharynx cancer**	2	8	-	-
** Testicular cancer**	1	4	-	-
** Cancer of unknown origin**	1	4	-	-
**Platinum**				
** Cisplatin**	21	84	-	-
** Carboplatin**	4	16	-	-
**Dose of platinum (mg)**				
** Cisplatin**	-	-	131.5	48–165
** Carboplatin**	-	-	626.5	450–750

### Pharmacokinetic assessment

**Figure 1:**
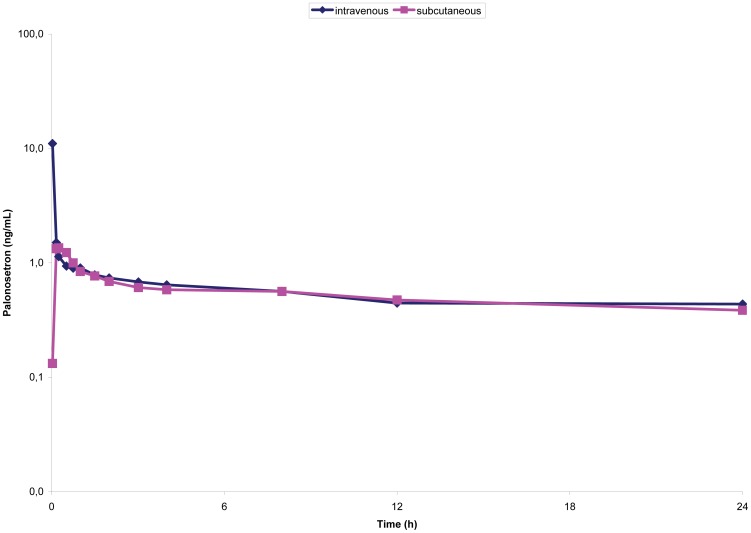
Palonosetron mean plasma levels (±SD) following a single 250 µg dose IV or SC (first 24 h, semilogarithmic graph).

**Figure 2:**
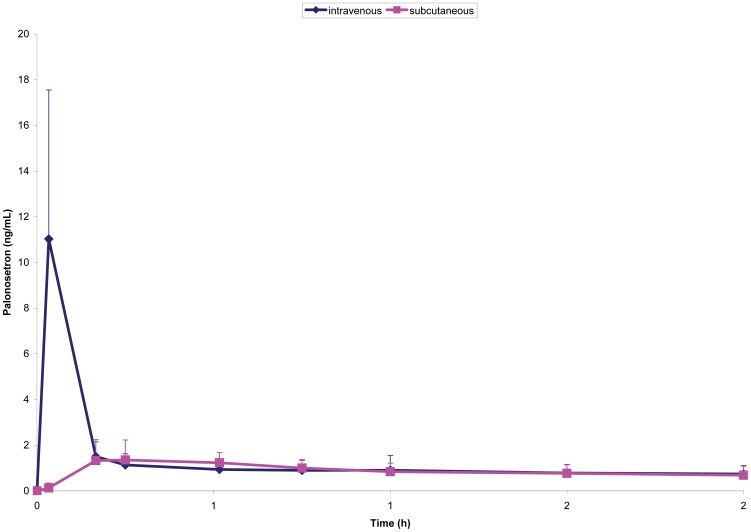
Palonosetron mean plasma levels (±SD) following administration of a single 250 µg dose IV or SC (first two hours).

Pharmacokinetic parameters are presented in table 2. Maximum plasma concentrations were observed right at the end of the IV infusion and 15 minutes after SC administration. C_max_ obtained after SC route was 15% (95% CI, 11–20%) of that one achieved by IV administration. Mean palonosetron plasma concentrations are presented on figures 1 and 2. AUC_0–24h_ and urinary elimination (20% dose administrated) were similar between both routes, indicating similar bioavailability with a relative F of 1.18 (118%). Other pharmacokinetic parameters, such as t_1/2_ and k_e_ were not statistically different.

**Table 2:** Pharmacokinetic characteristics of subcutaneous and intravenous palonosetron, compared by Student's t test for paired samples and Wilcoxon's test.

	IV	SC	p
	mean (± SD)	Mean (± SD)	
***AUC_0-24h_*** **(ng × h/ml)**	14.10±6.73	12.68±6.70	0.160
***C_max_*** **(ng/ml)**	11.88±7.38	1.91±1.09	<0.001
***t_max_*** ** (min)** 1	1 (1–10)	15 (10–32)	<0.001*
***k_e_ (h^−1^)***	0.095±0.117	0.075±0.061	0.527 *
***t_1/2_*** ** (h)**	12.71±10.21	14.68±9.79	0.527 *
***C_12h_*** **(ng/ml)**	0.487±0.292	0.459±0.289	0.671
***C_24h_*** **(ng/ml)**	0.415±0.206	0.414±0.235	0.365
***Ae_24h_ (%)***	19.48±9.99	22.24±8.50	0.660

IV: intravenous. SC: subcutaneous. AUC_0–24h_: area under the plasma drug concentration-time curve between 0 to 24 hours. n.s.s: non statistically significant. C_max_: maximum concentration. t_max_: time to maximum concentration. k_e_: elimination constant t_1/2_: half life. C: concentration. A_e_: amount of palonosetron eliminated by urine.

*: Wilcoxon's test.

1 : Median and range.

### Efficacy and toxicity assessment

From 25 patients evaluable for antiemetic efficacy, 11 (44%) reported no differences in antiemetic control between both alternatives, 6 (24%) had less emesis with SC palonosetron and 8 (32%) presented better control with the IV route. These differences were not statistically significant.

Nine patients (36%) reported constipation, (5 grade 1 and 4 grade 2). Other reported adverse events potentially related with study drug were headache (2), diarrhoea (2), hiccups (2), dizziness (1), skin rash (1) and bruise in the injection site (1). All these events were grade 1 and 2 and none were significantly more frequent with either administration route.

## Discussion

In this study, we have shown that palonosetron presents similar bioavailability when administered by either SC or IV route, confirming non-significant differences in AUC and urinary recovery between both routes. Therefore SC palonosetron seems a valid alternative to IV administration for control of emesis. This route could be of particular interest when conventional routes are difficult or impossible to use, for example, when heavy vomiting precludes oral intake or when IV administration is not possible in an outpatient setting. In addition, the SC route might be an interesting alternative for patients receiving oral chemotherapy that do not require IV medication.

Guidelines for management of emesis recommend the use of palonosetron with chemotherapy of moderate and high emetic potential (level 1, uniform consensus), and with chemotherapy regimens lasting over one day (level 2A, uniform consensus) [3], [4], [11]. We used a 250 µg dose of palonosetron since higher doses have not shown superior anti-emetic effect [12].

The observed t_1/2_ for the SC e IV routes were respectively 14.68 hours and 12.71 hours, within the range observed in previous studies [13], [14] Plasma palonosetron concentrations declined biexponentially after IV administration, with an initial rapid distribution phase followed by a slower elimination from the body. A C_max_ value of 5.63 ng/ml (SD = 5.48) has been previously reported after IV administration of 3 µg/kg (168–270 µg) of palonosetron over 30 seconds [12]. Considering differences in dose and sampling time, this is consistent with the C_max_ of 11.88 (SD = 7.38) ng/ml that we observed following IV administration. Absorption after SC administration of palonosetron was slow, and showed some influence of the absorption phase in the disposition of the drug. The maximum concentration was achieved 10–32 min after the dose, with an 85% reduction of C_max_ achieved after IV injection. In a previous study, a 15 minute IV infusion of 250 µg of palonosetron reduced decreased by 40% C_max_ as compared with a 30 second infusion [15]. It is unlikely that the differences in C_max_ observed between both routes can affect clinical efficacy, because the higher plasma concentrations after IV injection just lasted a short period of time, inferior to 5 minutes. In addition, since antiemetics are usually administered 30 to 60 minutes before chemotherapy, this difference is unlikely to affect clinical efficacy under a prophylactic point of view. Nevertheless, it could favor the IV route for treatment of established emesis, although, as previously mentioned, higher doses of palonosetron have not demonstrated higher clinical efficacy than lower doses.

This trial was not designed to compare the efficacy of both alternatives, and therefore, no definitive conclusions on this issue can be established based on our results. Yet, 44% of the patients reported no differences in control of emesis between both routes of administration, while 24% and 32% reported better control with SC and IV palonosetron respectively. These results were not statistically significant, and therefore suggest that SC administration might have similar antiemetic efficacy than the IV route, but additional studies will be necessary to confirm such preliminary observation.

Local toxicity was mild, with only 1 patient presenting a local reaction, which consisted on a bruise. Systemic toxicity mainly consisted on grade 1–2 headache and constipation. These adverse effects have previously been reported with 5-HT3 antagonists, including palonosetron. While the rate of headache is similar to what has previously been described [16], the proportion of patients presenting constipation is somewhat higher [17]. Nevertheless, this is probably explained by the fact that 4 patients presented previous constipation.

## Conclusion

SC administration of palonosetron has similar bioavailability than IV delivery. This is the first study that shows that SC palonosetron might be a valid alternative to IV administration. This new route of administration might be specially relevant for outpatient management of emesis in cancer patients and for oral chemotherapy regimens. Further studies are warranted to confirm the clinical value of SC palonosetron.

## Supporting Information

Checklist S1 **CONSORT Checklist.**
(DOC)

Protocol S1 **Trial Protocol.**
(DOCX)

Diagram S1 **CONSORT Flow Diagram.**
(DOC)
