# Supplementary material for: Randomized Pharmacokinetic Study Comparing Subcutaneous and Intravenous Palonosetron in Cancer Patients Treated with Platinum Based Chemotherapy
Source: PLoS One. 2014 Feb 27;9(2):e89747. doi: 10.1371/journal.pone.0089747 (PMC3937332; doi:10.1371/journal.pone.0089747)
Supplement: Protocol S1 — Trial Protocol. (DOCX) [file pone.0089747.s002.docx]

**CLINICAL TRIAL PROTOCOL**

**PAL/SC-IV-2008 Code**

**EUDRACT No.: 2008-002853-19**

**ABSORPTION AND SAFETY STUDY OF PALONOSETRON ADMINISTERED SUBCUTANEOUSLY IN CANCER PATIENTS.**

1st version: March 1, 2008

| Principal investigator |  | Dr. Belen Sádaba |
| --- | --- | --- |
| Institution where the study will |  | University Clinic of Navarra  Clinical Research Unit and Department of Oncology  Avda,Pio XII, No. 36  31008 Pamplona  Phone: 948 29 66 95  Fax: 948 29 65 00 |
|  |  |  |
| Sponsor |  | Science and Technology Institute of Navarra  Avda Pio XII, No. 53  31008 Pamplona  Phone: 948 17 67 48  Fax: 948 17 52 23 |
|  |  |  |
| Active |  | Palonosetron |
| Phase of study |  | Phase IV |

**CONFIDENTIAL**

**1.       Summary**

**- Type of Application**

A clinical trial of a medicinal product in new conditions.

**- Identification promoter**

Science and Technology Institute of Navarra

Avda Pio XII, No. 53

31008 Huarte - Pamplona

Phone: 948 17 67 48

Fax: 948 17 52 23Persona Contact: Dr. Isabel Gil Village.

**Two. - Title of clinical trial**

ABSORPTION AND SAFETY STUDY OF PALONOSETRON ADMINISTERED SUBCUTANEOUSLY IN CANCER PATIENTS.

**Three. - Protocol code**

EUDRACT: 2008-002853-19

Sponsor Code: PAL/SC-IV-2008

**April. - Principal Investigator.** **Address of your workplace.**

Dr. Belen Sádaba. Clinical Research Unit. University Clinic of Navarra (CUN). 31008 Pamplona.

**May. - Centers where testing is planned:**

Clinical Phase: Department of Oncology and Clinical Research Unit at the University Hospital of Navarra. Clinical Pharmacology.

Analytical phase: Pharmacokinetics Laboratory. Clinical Research Unit. University Clinic of Navarra.

**June. - Clinical Research Ethics Committee.**

The study requires the approval of the Ethics Committee of Navarra.

**July. - Name and qualification of the monitor.**

D. ---------------------------

**August. - Drug experimental and control: dose, dosage form, route of administration.**

- Experimental drug: ^®^ Aloxi (palonosetron)

Route of administration: subcutaneous (sc)

Pharmaceutical form of palonosetron hydrochloride ampoules

Dose: 250 μ g

Therapeutic group: A04AA

Laboratory Italfármaco

- Drug Control: Aloxi ^®^

Route of administration: intravenous (iv)

Pharmaceutical form of palonosetron hydrochloride ampoules

Dose: 250 μ g

Therapeutic group: A04AA

Laboratory Italfarmaco

Patients will be randomized to receive the first cycle via sc or iv. The second cycle will be managed by other means:

First stage: sc or iv administration, 30-60 minutes before chemotherapy administration.

Second stage: sc or iv infusion, 30 - 60 minutes before the administration of chemotherapy

**9. - Phase clinical trial.**

This is a Phase IV pharmacokinetic.

**10. - Main objective.**

Palonosetron explore pharmacokinetics when administered subcutaneously.

**11. - Design.**

Open, crossover, controlled trial in two phases. There shall be a minimum washout period of 14 days.

**12. - A disease or condition under study.**

Cancer patients undergoing chemotherapy requiring antiemetics.

**13. - Primary endpoint.**

Be assessed palonosetron pharmacokinetic parameters following:

Cmax = maximum concentration.

AUC _0-t_ =               area under the plasma concentration-time curve to the last extraction (time t).

tmax = time to reach Cmax.

**14. - Study population and number of volunteers.**

Cancer patients undergoing chemotherapy emetic. Will include a minimum of 25 evaluable patients (with pharmacokinetic evaluation SC and IV administration).

**15. - Duration of treatment.**

The regimen is administered for two consecutive chemotherapy cycles, so that the total duration of treatment and observation period is 6 to 10 weeks, depending on the timing of administration of chemotherapy.

**16. - Timetable and expected completion date**

Start Date:                                           September 2008

Recruitment end date:                             June 2009

Date of completion of treatment:               August 2009

**2.** **INDEX**

***1. SUMMARY***

***2.*** ***INDEX***

***3.*** ***GENERAL INFORMATION***

**3.1.** **Abbreviations**

**3.2.** **Administrative structure**

3.2.1.               Data relating to the Promoter

3.2.2.               Manager responsible for the development / control samples

3.2.3.               Monitor identification

3.2.4.               Data from the trial investigators.

**3.3.** **Study Report**

***4.*** ***JUSTIFICATION OF THE STUDY***

**4.1.** **Pharmacological aspects**

4.1.1.               Clinical Use

4.1.2.               Pharmacokinetics

4.1.3.               Adverse Reactions

4.1.4.               Precautions

**4.2.** **Evaluation of the risk / benefit**

***5.*** ***STUDY OBJECTIVES***

**5.1.** **Primary Objective**

**5.2.** **Secondary objective**

***6.*** ***TYPE OF TEST REPORT AND STUDY DESIGN***

**6.1.** **Type of clinical trial**

**6.2.** **Randomization**

**6.3.** **Study Periods**

**6.4.** **Design Justification**

***7.*** ***Population IN STUDIO***

**7.1.** **Anticipated number of subjects**

**7.2.** **Inclusion criteria**

**7.3.** **Exclusion criteria**

**7.4.** **Admission to the study**

**7.5.** **Identifying participants**

**7.6.** **Withdrawals and replacements**

***8.*** ***Study Treatments***

**8.1.** **Drugs**

**8.2.** **Dosing Schedule**

8.2.1.               Procedures medication administration

8.2.2.               Washout

8.2.3.               Precautions, antidotes

8.2.4.               Procedures blind

8.2.5.               Concomitant medication

8.2.6.               Adherence

**8.3.** **Administration of study medication**

8.3.1.               Labeling and distribution

8.3.2.               Storage and counting

***9.*** ***ASSAY DEVELOPMENT***

**9.1.** **Study Plan**

9.1.1.               Selection stage

9.1.2.               Intervention phase

9.1.3.               Final revision phase

**9.2.** **Handling of blood samples**

9.2.1.               Extractions

9.2.2.               Labeled

**9.3.** **Study duration**

***10.*** ***RESPONSE EVALUATION***

**10.1.** **Pharmacokinetic evaluation**

***11.*** ***Safety Assessment***

**11.1.** **Tolerance variables**

11.1.1.               Definition of an adverse event

11.1.2.               Defining an adverse event causality

11.1.3.               Procedures for recording adverse events

11.1.4.               Notification of adverse events

***12.*** ***STATISTICS***

**12.1.** **Statistical methods**

**12.2.** **Level of significance**

***13.*** ***ETHICAL AND REGULATORY***

**13.1.** **Clinical Research Ethics Committee (CEIC)**

**13.2.** **Guides and Documents**

**13.3.** **Information sheet and consent**

**13.4.** **Confidentiality of data**

**13.5.** **Patient Compensation**

**13.6.** **Insurance Policy**

**13.7.** **Notification to health authorities**

**13.8.** **Modifications to the protocol**

13.8.1.               Changes to the protocol

13.8.2.               Emergency Deviations Clinical Trial Protocol

**13.9.** **Withdrawal from the study**

**13.10.** **Interruption of study**

***14.*** ***QUALITY ASSURANCE AND CONTROL***

***15.*** ***INVESTIGATOR RESPONSIBILITIES AND PROMOTER***

**15.1.** **Responsibilities of the promoter**

**15.2.** **Responsibilities of the monitor.**

**15.3.** **Responsibilities of the investigator**

***16.*** ***DATA MANAGEMENT***

**16.1.** **Case reports of data (CDR)**

**16.2.** **Documents to guard the researcher**

**16.3.** **Study Archive**

**16.4.** **Monitorings**

**16.5.** **Final Report**

***17.*** ***PUBLICATION POLICY***

***18.*** ***REFERENCES***

**3.** **GENERAL INFORMATION**

***3.1.*** ***Abbreviations***

| AA | Adverse event |
| --- | --- |
| Competent Authority | Spanish Agency of Medicines and Health Products |
| AntiHBc | Virus core antibody B |
| AUC, AUC _0 -_ _∞,_ AUC 0-t | Area under the plasma concentration curve over time; extrapolated to infinity, to the last quantifiable extraction |
| BPC | Good clinical practice |
| BPL | Good Laboratory Practice |
| CEIC | Clinical Research Ethics Committee |
| Cmax | Maximum concentration |
| CRD | Notebook data collection |
| ECG | Electrocardiogram |
| VET | Specialty pharmaceutical advertising |
| EMEA | European Medicines Agency |
| FC | HR |
| FR | Respiratory |
| GGT | Gamma glutamyl transpeptidase |
| GOT (AST) | Aspartate amino transferase |
| GPT (ALT) | Alanine amino transferase |
| HBsAg | Australia Antigen B virus |
| HC | Medical record |
| BMI | í body mass index |
| mg, kg | Milligrams; kilograms |
| ml; L | Milliliters, liters |
| mm Hg | Millimeters of mercury |
| ND | Not determined |
| º C | Degrees Celsius |
| RD | Royal Decree |
| t _½_ | Elimination half-life |
| T ª | Temperature |
| TA, TAS, TAD | Blood pressure, systolic blood pressure, diastolic blood pressure |
| tmax | Time to reach Cmax |
| UIC | Clinical Research Unit |
| HIV | Human immunodeficiency virus |
| VSG | Erythrocyte sedimentation rate |

***3.2.*** ***Administrative structure***

*3.2.1.*        *Data relating to the Promoter*

Science and Technology Institute of Navarra

Avda Pio XII, No. 53

31008 Huarte - Pamplona

Phone: 948 17 67 48

Fax: 948 17 52 23

Contact: David Arana Ilardia

*3.2.2.*        *Manager responsible for the development / control samples*

Drug marketed in Spain by Italfármaco. Commercial preparation will be used, so that the processing is responsible for the laboratory itself marketer.

The inventory of medication is the responsibility of the principal investigator.

*3.2.3.*        *Monitor identification*

Monitor: --------------------------------

*3.2.4.*        *Data from the trial investigators.*

The clinical trial will be held between the Department of Oncology and Clinical Research Unit at the University Hospital of the University of Navarra.

Principal Investigator: Dr. Belén Sádaba. University Clinic of Navarra. 🕿 948 255400, 948 29 66 95

Researchers Collaborators: Dr. José Ramón Azanza Perea, Dr. Andrea Manubens Guarch, Dr. Urdaneta Matilde Abate, Dr. Almudena Gómez-Guiu Hormigos. Clinical Pharmacology Department of the University Clinic of Navarra. 🕿 948 296695. Dr. Alfonso Gúrpide, Dr. José Luis Pérez Gracia, Department of Medical Oncology and Radiotherapy 🕿 948 255400.

The analytical phase is performed in the Laboratory of Pharmacokinetics Clinical Research Unit at the University Hospital of the University of Navarra. The study director is Dr. Miguel Angel Martinez Ringer.

***3.3.*** ***Study Report***

The final report of the study is the responsibility of the Principal Investigator, the development of the analytical report shall be provided by the director of the analytical study.

**4** **JUSTIFICATION OF THE STUDY**

The study objective is to ascertain the pharmacokinetics of palonosetron after administration of the current formulation, Aloxi ^®,^ subcutaneously. In a previous study, the research team evaluated the possibility of administering granisetron subcutaneously, showing good results and adequate pharmacokinetic tolerance (Gúrpide 2007). Palonosetron is a more potent drug with a longer elimination half-life, which justifies a greater and more lasting. Therefore to assess the possibility arises to administer the drug subcutaneously.

Palonosetron used in the prevention of vomiting associated with moderately or highly emetogenic chemotherapy. At present there is a preparation for intravenous administration, but sometimes facilitate subcutaneous administration handling these situations. This is the interest of the research team, know the feasibility of administering the drug by this route, from the formulation for intravenous administration.

***4.1.*** ***Pharmacological aspects***

Antagonists 5-HT _3_ receptors are a family of important antiemetics to prevent emesis induced by cytostatic high emetogenic as cisplatin and lack of antidopaminergic activity. Have identified a range of different chemical structures with anti-5-HT3 receptors (ondansetron, granisetron, tropisetron, dolasetron, palonosetron etc ...). The differences between them lie in the chemical structure and the pharmacokinetic behavior, and its levels of effectiveness and side effect profile quite similar.

Currently antagonists 5-HT _3_ receptors are used as first line treatment in virtually all patients who receive highly emetogenic chemotherapy regimens (platinum regimens containing anthracyclines or other cytostatic high dose) in patients expected the emergence of side effects with metoclopramide or in those with inadequate response to previous treatment.

Palonosetron is an antiemetic agent, serotonin antagonist with high affinity for the 5HT _3_ receptor. It is the most potent antagonist in the treatment group. Prevents the occurrence of vomiting with a power between 2 and 93 times higher than that observed with ondansetron or granisetron.

*4.1.1.*        *Clinical Use*

Palonosetron is indicated for the prevention of acute nausea and vomiting associated with cancer chemotherapy moderately and highly emetogenic.

*4.1.2.*        *Pharmacokinetics*

Pharmacokinetics of palonosetron have been evaluated when administered parenterally. Bioavailability is not known in other ways.

Plonosetron shows linear kinetics μ between 0.3 and 90 g / kg (Eisenberg 2004).

It is widely distributed in the body, presenting a volume of distribution of 6.9 to 7, 9 l / kg (Sloltz 2004). The plasma protein binding is not high, about 62%.

The 40% of the administered drug is excreted in urine, 50% is converted by hepatic metabolism, mainly by the liver microsomal system. Mainly involved in the metabolism isoenzymes CYP2D6 and to a lesser extent, CYP3A4 and CYP1A2. Despite this involvement of cytochrome, has not been observed that the genetic polymorphism affecting the elimination of palonosetron.

The half-life of palonosetron is about 40 hours with a total clearance of 173 ml / min and the renal 53 ml / minute.

No change was observed in the pharmacokinetics in the elderly, renal or liver failure.

Palonosetron did not inhibit or induce cytochrome CYPP450. Nor has any suffered no interaction with drugs, inhibitors or inducers, administered concomitantly.

| **Pharmacokinetic parameters** | **Mean (CV)**  **n = 12** | **Mean (CV)**  **n = 11** | **Mean (**± **SD)**  **n = 11** |
| --- | --- | --- | --- |
|  | Hunt 2005 | Shah 2006 | Eisenberg 2004 |
| ***Dose mg*** | 0.25 | 0.25 | 3 μ g / kg |
| ***Infusion time (seconds)*** | 10 | 15 | 30 |
| ***AUC*** ***_0-24_*** **(ng** × **h / l)** | 8,900 (22%) | - | ± 8.570 4.220 |
| ***AUC*** ***_0 -_*** *_∞_* **(ng** × **h / l)** | - | 20,700 (25%) | ± 35,800 20,900 |
| ***Cmax*** **(ng / l)** | 1,130 (61%) | 919 (44%) | ± 5.630 5.480 |
| ***t*** ***_1/2_*** **(h)** | 42.8 (25%) | 37 (24%) | 56.4 ± 5.8 |

*4.1.3.*        *Adverse Reactions*

Palonosetron, a single dose administered before chemotherapy is well tolerated in general (Siddiqui 2004):

Side effects reported most often in connection with the administration of palonosetron were headache (9%) and constipation (5%).

*4.1.4.*        *Precautions*

Use cautiously in patients with constipation or signs of intestinal obstruction, because I could make symptoms worse.

Although no ECG changes observed with palonosetron, caution should extermarse when used concomitantly with drugs that prolong the QT interval of the ECG, as is done with other 5-HT _3_ antagonists.

***4.2.*** ***Evaluation of the risk / benefit* ratio**

We decided to use in this study a dose of 205 μ g, as is the usual recommended therapy in adults.

Patients remain hospitalized for 12 hours after drug administration, under the continued supervision of a medical team, which means they can control the adverse effects that may occur. The evaluation of the side effects with the administration of this drug and the dose to be administered indicate that it is likely to show significant tolerance problems.

There are no local tolerance data palonosetron, but after that observed in a previous study with granisetron and the few local problems after intravenous administration, suggest that even in this case there will be problems. In any case, the local tolerance is a secondary target, so that the patient will be especially guarded respect.

Therefore, based on available information and the design of the study, the sponsor and the investigator considered ethically acceptable trial.

This clinical trial is performed without direct individual benefit to the subjects participating in the study.

**5.** **STUDY OBJECTIVES**

***5.1.*** ***Primary Objective***

To explore Palonosetron absorption after subcutaneous administration, as compared with intravenous administration.

***5.2.*** ***Secondary objective***

To assess the local tolerance of palonosetron (VAS pain, skin changes).

**6.** **TYPE OF TEST REPORT AND STUDY DESIGN**

***6.1.*** ***Type of clinical trial***

Pilot clinical trial Phase I, single-center, open-label, single dose, with two periods of administration, controlled crossover study. It will randomize the sequence of administration of study medication.

***6.2.*** ***Randomization***

Subjects will be randomly assigned to each of the two treatment sequences.

The randomization number will be the one used to identify patients included in the study and will be reflected in the documents of the study.

Randomization was performed when deciding the administration of the first dose of medication. It will randomize the order of the route of administration under consideration by the list in Annex IX.

***6.3.*** ***Study Periods***

Selection phase: includes the 20 days before the start of the intervention phase. During this time, recruit volunteers, proving the suitability of the patient to participate in the study.

Study phase: consist sof two different treatment periods, separated by at least two weeks:

Both periods will be identical except for the route of administration the patient will receive, will depend on the randomization. Match management administration scheduled chemotherapy cycle.

Final review phase: includes the time from the completion of the intervention phase until the completion of the final visit, to be held in the next cycle.

***6.4.*** ***Design Justification***

The pharmacokinetic characteristics determine the estimated times of sampling, trying to define the likely tmax with subcutaneous administration and cover the entire absorption range.

The washout period is also conditioned by the terminal elimination half-life of palonosetron.

Since it is intended to assess bioequivalence, if not check if absorption occurs and what is the maximum concentration value is not considered appropriate to prolong the patient admission or request the removal of blood samples beyond the set range, which matches the patient's income for conrrespondiente chemotherapy.

Since there are no data on the absorption of subcutaneously palonosetron, pharmacokinetic analysis will be performed for the first four patients complete before continuing to include more patients. Special attention also to the local tolerance, but since it is a drug that is usually administered intravenously, with no evidence of phlebitis particular risk, it is considered that it should not have problems in this sense. Administration has been decided in the abdomen because it is necessary to administer a volume of 5 ml, since each vial contains the total dose diluted in this volume.

Using a dose of 250 μ g, which is the recommended sheet, in a single administration 30 minutes before administration of chemotherapy. Therefore maintaining marketing holder recommendation. In subcutaneous administration are to apply the same criteria.

**7.** **Population**

***7.1.*** ***Anticipated number of subjects***

The study will be conducted in 25 patients of both sexes. Since it is a crossover study in which each patient receives intravenous and subcutaneous administration, it is considered that 25 is a number sufficient to describe the pharmacokinetics of subcutaneously palonosetron, including bioavailability by this route.

***7.2.*** ***Inclusion criteria***

Patients must meet all of the inclusion criteria for participation in the study:

1. Patients requiring emetic chemotherapy of both sexes,
2. Age greater than 18 years,
3. ECOG ≤ 2.
4. Creatinine values ​​and normal liver function tests, considering an interval of 2 times the normal limits.
5. Adequate hematologic function defined by:
6. Absolute neutrophil count> 1,500 / mm ^3^ and WBC count> 3.000/mm ^3.^
7. Platelet count> 100,000 / mm ^3.^
8. Diagnosis of malignancy that needs treatment with chemotherapy with a moderate or high emetogenic potential (levels 3, 4 and 5 of Hesketh)
9. Have given written informed consent to participate in the study after having received all the information on it relating to design, objectives and possible risks resulting therefrom.
10. Ability to follow instructions and work together for the development of the study.

***7.3.*** ***Exclusion criteria***

None of the volunteers must submit any exclusion criteria, as it is referred to in the protocol:

1. Presence of infectious acute or chronic psychiatric disorders, clinically significant renal failure, at the discretion of the investigator.
2. Known hypersensitivity or intolerance to palonosetron.
3. Pregnant or lactating women with a positive pregnancy test.
4. Skin disorders that may interfere with the absorption of palonosetron, in the opinion of the investigator (edemas, inflammatory processes, thrombophlebitis ...).
5. Participation in another clinical trial with investigational products.

***7.4.*** ***Inclusion in the study***

After having given their consent in writing, once verified compliance with the inclusion and exclusion criteria, the patient was deemed eligible to participate in the study.

***7.5.*** ***Identification of participants***

Informed consent must be obtained in writing from each volunteer before any exploration.

The randomization number will be given after verifying that they have met all the inclusion criteria and none of the exclusion. This number will be determined by the scrambling, to be allocated before the administration of the first dose of study. This number will identify volunteers included in the study, and appear in all the documents of the trial.

The subjects will be identified also with 4 points, corresponding to the name (the first two) and two surnames.

Reference is made ​​to the full name and initials in identifying a list of subjects, which remain in the custody of the Principal Investigator. This list shall contain the identification of all patients signed informed consent, regardless of whether they were randomized after or not.

***7.6.*** ***Withdrawals and replacements***

Volunteers will be excluded from the study at their own request or by decision of the investigator when either of the following reasons:

intercurrent disease or AA if intolerable.

Violation of the foregoing restrictions.

Volunteer personal reasons for not continuing in the study.

Excluded subjects who leave the study for any of the above reasons will be replaced, to have a number of 25 evaluable patients, with complete pharmacokinetics of both routes of administration.

**8.** **Study Treatments**

***8.1.*** ***Drugs***

- Experimental drug: ^®^ Aloxi (palonosetron)

Route of administration: subcutaneous (sc)

Pharmaceutical form of palonosetron hydrochloride ampoules

Dose: 250 μ g

Therapeutic group: A04AA

Laboratory Italfarmaco

- Drug Control: Aloxi ^®^

Route of administration: intravenous (iv)

Pharmaceutical form of palonosetron hydrochloride ampoules

Dose: 250 μ g

Therapeutic group: A04AA

Laboratory Italfarmaco

Patients will be randomized to receive the first cycle via sc or iv. The second cycle will be managed by other means:

Both medications will be supplied by the promoter. It is the responsibility of the investigator administer the study medication according to the randomization code.

The medication is stored correctly labeled, according to the conditions proposed by the developer.

***8.2.*** ***Dosing Schedule***

This is a single dose study. Patients will receive on two separate occasions a single palonosetron dose of 250 μ g, once the administration is subcutaneous, intravenous in the other. The order of the route of administration will be allocated according to a randomization table.

*8.2.1.*        *Procedures medication administration*

The administration of medication will be as follows:

- First cycle: sc or iv administration, 30-60 minutes before chemotherapy administration.

- Second cycle: sc or iv infusion (the opposite way than first cycle), 30 - 60 minutes before the administration of chemotherapy

The order of the route of administration depends on the randomization table. The administration will be in the abdomen when subcutaneously. In the case of intravenous administration, the same route may be used in place for administration of chemotherapy or other medications that the patient should receive as their medical criteria.

Intravenous administration will be performed quickly, in 30 seconds, as described in the Product data sheet.

Subcutaneous administration will also perform at this time, in the abdomen.

*8.2.2.*        *Washout*

Has established a washout period between the two administrations of 14 days, which is considered sufficient as the pharmacokinetic data observed palonosetron in the literature.

*8.2.3.*        *Precautions, antidotes*

Available emergency equipment and standard operating procedure for the treatment of non-specific reactions (allergy, anaphylactic shock). At all times there will be a doctor reachable to respond to unforeseen developments.

*8.2.4.*        *Procedures blind*

It is an open, not hidden treatments either the researchers nor the volunteers, but for those responsible for drug quantification analysis.

*8.2.5.*        *Concomitant medication*

The investigator assessed the impact on the study in the event that any of the volunteers had to receive some treatment concomitantly.

Will try the two periods of administration of study medication are similar in concomitant medication.

*8.2.6.*        *Adherence*

Adherence will be secured by the research team, who directly administer the medication. Log the exact times of drug administration and removal of samples.

***8.3.*** ***Administration of study medication***

*8.3.1.*        *Labeling and distribution*

The products under investigation will be provided by the developer in sufficient quantities, they will get it directly from the manufacturer. The medication does not require any prior manipulation, is directly administered undiluted.

Upon receipt of trial medication, the Pharmacy shall duly completed (signed and dated) to the promoter acknowledgment of medication.

*8.3.2.*        *Storage and counting*

The records and inventory of the samples must be stored by the Pharmacy Service Center or by the investigator and will follow the following rules:

The investigator should retain the test samples at the pharmacy or store them in a safe and accessible only to persons authorized to dispense investigational drugs.

The inventory will be conducted by the researcher or pharmacist or other person designated by the former.

The inventory record shall detail the material received, and clearly indicated when and what subject was dispensed.

The register shall indicate the quantity and description of the research material that has at any time during the clinical trial.

At the end of this trial the researcher agrees to make the final inventory of the material and enter the result thereof in one form ″ ″ Medication count. The investigator or the pharmacist must return all materials to the promoter and other continent whether they are empty as if they still contain test samples.

The researcher agrees not to provide study drugs to anyone except those named as collaborating investigators and study volunteers.

**9.** **ASSAY DEVELOPMENT**

***9.1.*** ***Study Plan***

*9.1.1.*        *Selection stage*

In the 20 days before the start of the intervention phase of the study will be the selection of volunteers. This phase will recruit 25 patients will be informed of the design, objectives and possible risks of the study. Each patient will have the opportunity to ask any questions you want to research. Before any test is obtained written informed consent.

They perform a complete medical history that includes:

• Detailed anamnesis.

• Complete physical examination with assessment of functional status (ECOG scale).

• Registration of drugs administered to the patient at the time of inclusion.

• Laboratory tests:

Hematology: CBC.

Biochemistry: blood: cre atinina, urea, SGOT (AST), SGPT (ALT), GGT, total bilirubin, electrolytes

In case the patient has already been subjected to this analysis in the 10 days prior to administration of the medication can not be repeated.

With all these data verify the eligibility of patients to participate in the study.

*9.1.2.*        *Intervention phase*

The intervention phase will consist of two periods in which the patient will receive the study medication. Each will have a minimum duration of 12 hours, separated by at least two weeks washout period. In each volunteer will receive a dose of palonosetron 250 μ g intravenously or subcutaneously, according to the randomization code. Before the second administration of the drug is verify that the patient has adequate hepatic and renal function.

Will place a peripheral venous independent pathway by the patient receive chemotherapy and palonosetron, through which the blood samples drawn for determination of the drug.

They undertake every single day of intervention:

- Determination of the SBP and DBP, FC and temperature in a sitting position before administration of the study medication.

Blood drawn at baseline (5 ml).

Assessment of pain by visual analog scale.

After drug administration was performed:

Blood samples (5 ml) at the following times: end of infusion, 10 - 15 - 30 - 45 - 60 mins, 1,5 - 2 - 3 - 4 -8 to 12 hours. If income permits 24 hours.

Collection of urine during the 12 hours of study.

Subjective assessment of pain using a visual analog scale at the time: 1 to 4 and 8 hours.

Physical examination of the administration at 1, 4 and 8 hours after administration.

Each extraction with 5 ml of blood obtained. Samples were extracted in 5ml tubes through an Abbocath ® or a similar device placed in the arm contralateral to the administration of chemotherapy.

The track maintenance extraction will take place in optimum conditions by introducing, after each drawing 5 ml of a solution prepared with 1 ml of 1% sodium heparin, and 9 ml of 0.9% NaCl through the device.

Drawing the samples will be transferred into a glass with ice to the Laboratory of Clinical Pharmacokinetics Clinical Research Unit, where they are processed and frozen until analysis. The manipulation of the samples, the biological matrix and the storage temperature will be confirmed by the technician in charge of the analysis.

The determination of plasma concentrations of palonosetron be performed by HPLC.

*9.1.3.*        *Stage final review*

At least two weeks after the last drug administration, before the next cycle, check the patient's situation, using the same criteria as the selection period.

***9.2.*** ***Handling of blood samples***

*9.2.1.*        *Extractions*

After administration of palonosetron, there will be blood samples (5 ml) at the following times: end of infusion, 10 - 15 - 30 - 45 - 60 mins, 1,5 - 2 - 3 - 4 -8 to 12 hours. If income permits 24 hours.

Blood will be drawn from the vein. EDTA tubes are used (5 ml).

Samples were centrifuged at 3500 rpm for 10 minutes at 4 ° C within 30 minutes after removal. The resulting plasma was separated into two aliquots for determination of palonosetron, and freeze at -70 ° C until shipment to the laboratory for analysis. Palonosetron determining aliquots of plasma will be by high resolution liquid chromatography with mass spectrometer detection (LC / MS). The conditions of sample handling can be modified depending on the analysis technique, which is in development.

*9.2.2.*        *Labelling*

Samples for the determination of palonosetron is labeled with the following information:

-           Line 1:               Name of the active ingredient (palonosetron).

-           Line 2:               study identification code. Route of administration. Aliquot (original or duplicate).

-           Line 3:               Number of subject randomization. Initials. Period administration.

-           Line 4:               The time of extraction.

***9.3.*** ***Duration of study***

The clinical trial will consist of a selection phase, the intervention phase (or treatment) and final review visit.

In the selection phase, each subject will be selected for participation in the study during the 20 days prior to drug administration. Intervention phase include two treatment periods with a minimum of washing including 14 days, calculated taking into consideration the mean elimination half-life of palonosetron described in the literature (t1 / 2 = 40 hours). Each patient will receive the medication twice. They collected blood and urine samples as specified. The final review is held after the second treatment cycle.

**10.** **RESPONSE EVALUATION**

***10.1.*** ***Pharmacokinetic evaluation***

The quantification will be palonosetron concentrations in the Laboratory of Pharmacokinetics. Clinical Research Unit. University Clinic of Navarra. The Center is certified in Good Laboratory Practices.

With plasma concentration data at different times are calculated palonosetron pharmacokinetic parameters.

Primary parameters are considered in this study:

Cmax = maximum concentration. Its value will be extracted from the experimental data.

AUC _0-t_ = Area under the plasma concentration curve from time 0 to the last quantifiable concentration (time t) calculated by the trapezoidal method.

tmax =               time to reach Cmax. Its value will be extracted from the experimental data.

Security variables are:

VAS at different measurement times (baseline, and after the administration at 1, 4 and 8 hours)

Exploration of the administration at the same times.

The safety variables also include the description of the adverse events reported by patients or alterations in tests.

**11.** **Safety Assessment**

Patients remain in contact with the Center's staff, having received the medication under supervision and being evaluated in conjunction with each of the blood samples. A doctor will be available throughout the study at any time.

***11.1.*** ***Tolerance variables***

An evaluation of all adverse events reported by patients or observed by the research team, including abnormal laboratory findings.

Each volunteer will be asked in general: Do you see any difference in your physical condition from the previous to the administration of the drug?, Coinciding with each blood sample. Any AA referred spontaneously or as a result of the research question will be recorded in the medical record of the volunteer, specifying the time of onset, course, duration, intensity, necessary treatment and medication relationship. Upon completion of the survey will be repeated physical examination and laboratory tests each volunteer.

*11.1.1.*     *Definition of an adverse event*

An AE is any undesirable experience occurring or worsening during the course of the study, regardless of causal relationship to study medication. In addition, all events occurring in connection with the study before or after the clinical period, ie between the screening visit and the visit of final review, must be valued as an AA. Adverse events are not considered drowsiness, sedation, gait instability or standing, but its appearance is collected in the same manner as other adverse events.

The following effects are classified as AA:

Worsening (change in the nature, intensity or frequency) of the conditions at baseline.

Deterioration of the subject due to primary disease.

Intercurrent disease.

Drug Interactions.

Events related or possibly related to concomitant medication.

Significant abnormal laboratory values ​​and significant deviations from the reference values, but within the normal range, which the investigator considered clinically important.

The AA will be recorded on a sheet of AA history and classified as mild, moderate or severe, according to the following definition:

Minor: not cause limitation of usual activities. The subject may experience a slight discomfort.

Moderate : cause some limitation of usual activities. The subject may experience discomfort.

Severe : inability to perform usual activities. The subject may experience intolerance, discomfort or pain.

A grave is a AA AA is:

Fatal.

Threatening to life.

Produce permanent disability.

subject requires hospitalization or prolongation of the same.

Produce congenital anomaly.

Cancer.

Overdose or intoxication.

Any AA that, in the opinion of the investigator, is considered serious and is not covered by the above.

An unexpected AA experience is defined as described not in terms of nature, severity or frequency in the investigator's brochure ..

*11.1.2.*     *Defining an adverse event causality*

The investigator will determine the relationship of any AA with study medication according to the following criteria (according to "Modified to the classification of Karch / Lasagna, Journal of the American Medical Association 234:1236-1241, 1975"):

*Short* : the reaction is with a reasonable time sequence after drug administration. The effect had previously been referred to or is an expected response to the study drug, confirming an improvement after the reduction or suspension of the drug and recurrence of effect after readministration of the same, with no apparent alternative etiology.

*Probable* : Reaction appears reasonable time sequence after drug administration. The effect has already been referred to previously or is an expected response to the study drug. Improvement occurs after reduction or discontinuation of treatment, and any other etiology is unlikely or less likely.

*Possible* : The reaction appears reasonable time sequence after drug administration. The effect had previously been referred to or is an expected response to the study drug. There is a possible alternative that may be responsible etiological AA.

*Unlikely* : the reaction does not appear with a time sequence after drug administration, or if exists, it is remote. The effect is not an expected response, or known to the study drug. There is a possible alternative that may be responsible etiological AA.

*Unrelated* : the AA is due to an underlying disease or concurrent or the effect of another drug and is not related to study medication (not to be related temporary or most likely alternatives exist).

The parameters of clinical and laboratory tolerance will be evaluated descriptively and differences using a Student t test (paired data), indicating hematological and biochemical parameters (See Annex IV for reference values) and BP, HR, RR and T ª, will be discussed and AA abnormal values. If any abnormality is observed, will convene again to volunteer to repeat this determination and this will be followed up until all changes are considered as clinically relevant.

*11.1.3.*     *Procedures for recording adverse events*

All AA that occur during administration of study treatment, either spontaneously reported by the subjects or observed by the investigator, are (in the opinion of the investigator) related or not, must be picked up in the history and transcribed the AA page contained in the CRD. The researcher also determined the relationship between AA and study drugs and recorded in the appropriate section of the notebook.

*11.1.4.*     *Notification of adverse events*

The researcher informed all AA promoter that occur during the study, regardless of their relationship to the study drugs.

A serious or unexpected AA or not related to study medication ICT must be informed within 24 hours of becoming aware of it.

After disclosure, the investigator shall complete and sign a report for the written notification to the promoter.

The researcher, as delegated by the Promoter, communicate to the competent authority within the AA up to 7 days in the case of AA involving fatal or life-threatening (those not been for immediate therapeutic intervention would have meant the death of the patient ). If not had all the information it can be completed within an additional 8 days. Other unexpected serious AEs be reported within 15 days.

Reports on the course which the subject in relation to the AA should be made until it is gone or the clinical situation has stabilized and presented to the developer. If required, additional information will be provided.

It is the responsibility of the Promoter AA reporting serious and unexpected to the Clinical Research Ethics Committee, the Spanish Agency for Medicines and Health Products and Pharmacovigilance Center of Navarre. On the relationship between developer and researcher, the first delegates to the researcher communicating these AA to health authorities and CEIC.

**12.** **STATISTICS**

***12.1.*** ***Statistical methods***

The pharmacokinetic and statistical analysis of the data will be performed by UIC in the University Clinic of Navarra.

Pharmacokinetic analysis was performed on the data of concentrations of all evaluable volunteers completed the study as set forth in the protocol: pharmacokinetic profile with both formulations. The following pharmacokinetic parameters of palonosetron, will be determined for each subject following administration of each treatment:

-Cmax = maximum concentration,

-AUC _0-t_ = area under the curve versus time to the last quantifiable sample (time t), calculated using the trapezoidal method.

-Tmax = time of Cmax is reached,

-The elimination half-life, t _1/2_ , will be determined by the following expression:

t _½_ = ln 2 / k _e_

There will be a descriptive analysis of palonosetron pharmacokinetic parameters after subcutaneous administration.

Bioavailability is calculated in this way by calculating the ratio of AUC _0-t_ obtained after subcutaneous and intravenous administration.

Other parameters (analytical data, blood pressure) were analyzed using Student t test for paired data or ANOVA as appropriate, after having determined that the conditions for applying these tests. In the case of non compliance will be analyzed using appropriate nonparametric tests (Wilcoxon, Friedman).

***12.2.*** ***Level of significance***

The level of statistical significance was set at *p* <0.05.

**13.** **ETHICAL AND REGULATORY**

***13.1.*** ***clinical research ethics committee (CEIC)***

This study may be initiated only after written approval from the Ethics Committee of Navarre and the sponsor and investigator have received a copy of such approval.

***13.2.*** ***Guides and Documents***

This study will be conducted according to "Note for Guidance on Good Clinical Practice" (CPMP/ICH/135/95 of May 1, 1996), Royal Decree 223/204 of February 2004 and the Declaration of Helsinki, revised Edinburgh, 2000.

***13.3.*** ***Information sheet and consent***

The researcher obtained from all subjects participating in the study written informed consent (Annex VIII). The investigator is responsible for obtaining proper informed consent. Shall:

Explain to each subject the nature, objectives, potential risks / benefits and duration of the clinical trial and any additional test is performed.

Inform each individual from participation in the research project is not mandatory and you can withdraw at any time without prejudice.

inform the subject of the medical history may be verified by the persons appointed by the promoter and / or Control Authorities.

Inform the subject that there is a liability policy in case you suffer an injury due to the trial medication.

Informed consent should be obtained after the delivery of the "Patient Information Sheet" and before any study-specific procedure. Registration of informed consent will be inspected by persons appointed by the developer and by health authorities and control as required.

***13.4.*** ***Confidentiality of data***

In order to ensure the confidentiality of the trial data, only have access to the same promoter, the monitor, the researcher and his team, the Clinical Research Ethics Committee which protects the assay and the relevant health authorities.

The content of the Journal of Data Collection (CRD) and the documents generated during the study will be protected from unauthorized uses by outsiders to research, and therefore, will be considered strictly confidential and will not be disclosed to third parties other than those specified in the previous paragraph. These documents shall be identified by the scrambling code, so that no identifying information of participants leaving the clinical site.

It will in any case law on personal data protection, law 15/1999.

***13.5.*** ***Patient Compensation***

No compensation is provided to the patient for their participation in the study.

***13.6.*** ***Insurance Policy***

The sponsor should ensure the safety of the subjects participating in the study and indemnify in the event of damage as a result of study drug administration. The developer has a liability policy with the company hired HDI, which will cover any type of risk arising from the study (Annex V) for both subjects and for participating physicians (provided there is no negligence on the part of the physician or your computer), as regulated in RD: 223/2004 clinical trial.

***13.7.*** ***Notification to health authorities***

This test requires an application to the Hon Mr Director, Spanish Agency for Medicines and Health Products (Competent Authority) for approval. The study must be approved by the Competent Authority before they can start.

***13.8.*** ***Modifications to the protocol***

*13.8.1.*     *Changes to the protocol*

The protocol changes are made only when such changes have been agreed in writing and signed by the principal investigator and the sponsor. Be informed of any relevant modification to CEIC and the Competent Authority. If the change represents a greater risk to the subjects, must be approved by the Ethics Committee and the Competent Authority.

The changes to the protocol may represent changes in the informed consent of all volunteers, both prospective and already included. Volunteers must sign a new consent if these changes occur.

*13.8.2.*     *Emergency Deviations Clinical Trial Protocol*

Protocol Deviations shall only be carried out in the event of an emergency in a subject requires it, and that deviation will be only for that subject. The researcher is of the emergency service will occur in telephone contact with the developer as soon as possible. The researcher will then decide whether the volunteer who has deviated from the protocol or not to continue in the study. In the HC and the CRD will describe the deviation of the Protocol, stating the reasons that motivated it.

***13.9.*** ***Withdrawal from the study***

Volunteers will be informed that they can withdraw at any time. This removal may occur by any of the following reasons:

Will the participant.

Significant breach protocol.

Intercurrent disease that interferes with the development of the study.

Occurrence of adverse events or other situations that, in the opinion of the investigator, pose a risk to the volunteer.

Appearance of exclusion criteria for the study

The withdrawal will be documented in the medical record, stating the reasons. Should be conducted, as far as possible, a final review visit when you have received at least one of the study medications.

He will replace retired volunteers, if is compromised the validity of the study, ie the number of evaluable volunteers is less than 25.

***13.10.*** ***Interruption of study***

The sponsor may stop the study at any time.

In case of serious AEs or other unforeseen circumstances, the principal investigator may discontinue the full study, ethical or medical reasons, after consultation with the sponsor.

**14.** **QUALITY ASSURANCE AND CONTROL**

Quality control of the data collected in the CRD will be assured by the test monitor as monitoring procedures.

The monitor tracking control trial protocol.

The assay may be inspected by the regulatory authorities. In this case, inspectors can make a visit to the research center to assess compliance with GCP.

Authorized personnel may perform audits on the test site before, during or after completion of the study in close connection with the investigator and the monitor and / or clinical research associate.

**15.** **INVESTIGATOR RESPONSIBILITIES AND PROMOTER**

***15.1.*** ***Responsibilities of the promoter***

The responsibilities of the developer include:

Establish and maintain a system of quality assurance and control, with written standard operating procedures, so that the tests are conducted and data generated, documented and reported in accordance with the protocol, the standards of good clinical practice RD 223/2004.

sign, along with the corresponding investigator, the protocol and any amendments thereto.

Select the most appropriate investigator according to their qualification and facilities available, and ensure that it will perform the study as specified in the protocol.

Provide basic and clinical information available for the product in research and update throughout the trial.

Request the opinion of the Clinical Research Ethics Committee and approval of the Competent Authority, as well as giving information and obtain approvals to proceed, subject to communication to the regions, if the amendment or protocol violation or interruption trial, and the reasons for it.

Provide free investigational medicinal products, ensure that they have met the standards of good manufacturing practice and that the samples are properly packaged and labeled. It is also responsible for the storage of samples and their manufacturing and testing protocols, registration of samples delivered and to ensure that in the center where the test is done there will be a proper procedure for handling, storage and use of such samples.

Designate the monitor that will monitor the progress of the trial.

Inform the health authorities, researchers and the Clinical Research Ethics Committees involved in the trial of suspected unexpected serious adverse reactions.

Provide the investigator and the Clinical Research Ethics Committee, immediately, any information of importance to have access during the test.

To provide financial compensation to the subjects in case of injury or death associated with the assay. Provide legal and economic research coverage in these cases except where the harm is the result of negligence or malpractice of the researcher.

agree with the researcher's obligations regarding data processing, reporting and publication of results. In any case, the developer is responsible for preparing the final reports or partial trial and communicate them to the appropriate parties.

The sponsor shall have one point of contact, where trial subjects may obtain further information on this, which may delegate to the researcher.

***15.2.*** ***Responsibilities of the monitor.***

The monitor is the main means of communication between the sponsor and the investigator. His responsibilities include:

Work according to standard operating procedures of the promoter, visit the investigator before, during and after testing to verify compliance with the protocol, ensuring that the data are recorded correctly and completely, and make sure that it has informed consent was obtained from all subjects before inclusion in the trial.

Ensure that researchers and the center where the research will be suitable for this purpose during the period of the trial.

Ensure that both the principal investigator and his collaborators have been informed properly and always ensure fast communication between researcher and developer.

Verifying that the investigator meets the protocol and all amendments approved.

Check that the storage, distribution, return and documentation of investigational drugs is safe and appropriate.

Submit reports to the sponsor monitoring visits and all relevant contacts with the investigator.

***15.3.*** ***Responsibilities of the investigator***

The researcher will be responsible for following the rules below:

To agree and sign together with the promoter assay protocol.

depth knowledge of the properties of investigational medicinal products.

Ensure that informed consent is collected pursuant to the provisions of Royal Decree 223/2004.

collect, record and report the data correctly and ensure its accuracy.

Immediately notify the serious or unexpected adverse events to the sponsor.

Ensure that all concerned respect the confidentiality of any information about the test subjects and the protection of their personal data.

Report regularly to the Clinical Research Ethics Committee of the progress of the trial.

co-responsible with the promoter of the final report of the trial, giving his agreement with his signature.

**16.** **DATA MANAGEMENT**

***16.1.*** ***Notebooks of data collection (CDR)***

The CRD (Annex I) shall be prepared by the research team transcribed the data from the original papers constituting the volunteer's medical history. The original CRD sent to the promoter and another copy will remain in the center. The CRD will be recorded in indelible black ink all the details required by the developer, along with all the significant additional details. The investigator shall sign and date the CRD to ensure its authenticity and accuracy. The CRD can be completed by any authorized person whose signature is recognized. Any change will be made visible and bear the initials of person making the correction and the date on which it is carried out. The CRD filled will be reviewed during the monitoring visit.

Any result outside the normal range will be duly discussed.

***16.2.*** ***Documents to guard the researcher***

The investigator will retain all original documents of medical records and a copy of all the CRD and the list of participants to identify subjects for 15 years or until the promoter tells you otherwise. This documentation will not be destroyed without the written consent of the Promoter of the study.

***16.3.*** ***Archive study***

ICT maintain a Studio Master File during product life.

***16.4.*** ***Monitorings***

The promoter will assign staff to clinical trial monitoring. Among its functions appear to help the investigator and the sponsor in maintaining data fully legible, well organized and easy to retrieve. Also explain and ensure that the researcher understands and interprets all SOPs for the promoter and the relevant provisions relating to the clinical evaluation of an investigational drug. It will also ensure that the Protocol is understood correctly, and inform the responsibilities and the validity of the data.

***16.5.*** ***Final Report***

The team will prepare a report suitable for submission to the relevant authorities.

**17.** **PUBLICATION POLICY**

All information provided concerning the drug and the clinical trial is confidential and belongs to the promoter. The researcher can use this information only for matters related to the trial. It will also have an obligation to provide all data generated in the study to the promoter.

All results, data and information developed, generated and derived from this clinical trial will be the sole property of the promoter and consequently the researcher agrees to treat such information as confidential and secret as mentioned above. The researcher agrees not to disclose the information in any way without the prior written consent of the promoter.

**18.** **REFERENCES**

Eisenberg P, MacKintosh FR, Ritch P, Cornett PA, Macciocchi A. Efficacy, safety and pharmacokinetics of palonosetron in patients Receiving highly emetogenic cisplatin-based chemotherapy: a dose-ranging clinical study.Ann Oncol. 2004, 15 (2): 330-337.

Gurpide A, Sadaba B, Martin-Algarra S, Azanza JR, Lopez-Picazo JM, Ringer MA, Cabello JP, Gil-Village I, Cross S, Fernandez Gallego V, Reyna C, Olier Garate C, Blanco-Prieto MJ , Ceballos J, Garcia-Foncillas J, Perez-Gracia JL. Randomized crossover pharmacokinetic evaluation of subcutaneous versus intravenous granisetron in cancer patients platinum-based TREATED WITH chemotherapy.Oncologist. 2007, 12 (9): 1151-1155.

Hunt TL, Gallagher SC, Cullen MT Jr, Shah AK. Evaluation of safety and pharmacokinetics of consecutive multiple-day dosing of palonosetron in healthy subjects.J Clin Pharmacol. 2005, 45 (5): 589-596.

Shah A, T DeGroot, Apseloff G. Pharmacokinetic evaluation and safety profile of a 15-minute versus 30-second infusion of palonosetron in healthy subjects.J Clin Pharmacol. 2006, 46 (10): 1139-1145.

Siddiqui MA, Scott LJ.Palonosetron.Drugs. 2004, 64 (10): 1125-1132, discussion 1133-1134.

Stoltz R, Parisi S, Shah A, Macciocchi A. Pharmacokinetics, metabolism and excretion of intravenous [L4C]-palonosetron in healthy human volunteers.Biopharm Drug Dispos. 2004, 25 (8): 329-337.

hem
