# Supplementary material for: Randomized Pharmacokinetic Study Comparing Subcutaneous and Intravenous Palonosetron in Cancer Patients Treated with Platinum Based Chemotherapy
Source: PLoS One. 2014 Feb 27;9(2):e89747. doi: 10.1371/journal.pone.0089747 (PMC3937332; doi:10.1371/journal.pone.0089747)
Supplement: Diagram S1 — CONSORT Flow Diagram. (DOC) [file pone.0089747.s003.doc]

**CONSORT 2010 Flow Diagram**

**Allocation**

**Analysis**

**Follow-Up**

**Enrollment**

Assessed for eligibility (n= 35)

Excluded (n= 6)

♦  Not meeting inclusion criteria (n=6)

Analysed (n= 13)
♦ Excluded from analysis (give reasons) (n= 2)

- anaphylactic shock during administration of paclitaxel (1)
- volunteer decision to leave the study (1)

Lost to follow-up (give reasons) (n= 0)

Discontinued intervention (give reasons) (n= 2)

Allocated to subcutaneous palonosetron followed by intravenous palonosetron (n=15)

♦ Received allocated intervention (n= 15)

♦ Did not receive allocated intervention (give reasons) (n= 0)

Lost to follow-up (give reasons) (n= 0)

Discontinued intervention (give reasons) (n= )

Allocated to intravenous palonosetron followed by subcutaneous palonosetron (n=14)

♦ Received allocated intervention (n= 14)

♦ Did not receive allocated intervention (give reasons) (n= 0)

Analysed (n= 12)
♦ Excluded from analysis (give reasons) (n=2)

- death due to disease progression (1)
- chemotherapy related neutropenia (1)

Randomized (n=29)
